# High-Resolution Northern Blot for a Reliable Analysis of MicroRNAs and Their Precursors

**DOI:** 10.1100/tsw.2011.11

**Published:** 2011-01-05

**Authors:** Edyta Koscianska, Julia Starega-Roslan, Katarzyna Czubala, Wlodzimierz J. Krzyzosiak

**Affiliations:** Laboratory of Cancer Genetics, Institute of Bioorganic Chemistry, Polish Academy of Sciences, Poznan, Poland

**Keywords:** northern blotting, single-nucleotide resolution, miRNA, pre-miRNA, miRNA length heterogeneity

## Abstract

This protocol describes how to perform northern blot analyses to detect microRNAs and their precursors with single-nucleotide resolution, which is crucial for analyzing individual length variants and for evaluating relative quantities of unique microRNAs in cells. Northern blot analysis consists of resolving RNAs by gel electrophoresis, followed by transferring and fixing to nylon membranes as well as detecting by hybridization with radioactive probes. Earlier efforts to improve this method focused mainly on altering the sensitivity of short RNA detection. We have enhanced the resolution of the northern blot technique by optimizing the electrophoresis step. We have also investigated other steps of the procedure with the goal of enhancing the resolution of RNAs; herein, we present several recommendations to do so. Our protocol is applicable to analyses of all kinds of endogenous and exogenous RNAs, falling within length ranges of 20–30 and 50–70 nt, corresponding to microRNA and pre-microRNA lengths, respectively.

### INTRODUCTION

MicroRNAs (miRNAs) are well-known post-transcriptional regulators of gene expression found in all multicellular organisms. These tiny (~20 nt) RNAs regulate many physiological processes, and the deregulation of miRNA expression in human cells is linked to numerous diseases[1,2,3,4,5,6]. These facts explain why so much effort has been exerted toward reliable miRNA detection methods. Many attempts have been made to develop highly sensitive methods of miRNA analyses and to improve the existing methods to make miRNA characterization more accurate. The detection of miRNAs is more challenging than the detection of larger RNAs. This is not only due to their small size, but also to the sequence similarity frequently observed among members of miRNA families and to the low expression levels of most miRNAs.

Current methods for miRNA detection, including their advantages and drawbacks, as well as future perspectives of miRNA analyses have been extensively discussed[7,8]. Briefly, the existing methods are predominantly based on hybridization, which may occur in a solid phase (e.g., northern blotting[9,10], microarray technologies[11,12], real-time PCR[13,14], electrochemical methods[15]) or a solution phase (e.g., luminescence- [16], fluorescence- [17], colorimetric- [18], and enzyme-based miRNA detection[19]). The most serious drawbacks of these methods include limited sensitivities, lengthy procedures, inability to convert to a multiplex format, and difficulties with signal quantification.

The most standardized and widely used miRNA detection method is northern blotting. This method is considered the "gold-standard" in miRNA detection and every effort is being made to improve upon this method. In this protocol, we refine northern blotting to the highest possible resolution in order to detect both miRNAs and pre-miRNAs, as well as to allow their reliable quantification. This is an important issue in studies on miRNA biogenesis and function. High precision is required when determining the cellular levels of individual length variants of specific miRNAs because these variants may regulate different sets of targets[20,21].

### Development of the Protocol

Our high-resolution northern blot protocol was successfully used for the detection of miRNA and pre-miRNA variants in human cells. The study was designed to evaluate the frequency of length heterogeneity of miRNAs and pre-miRNAs, which arises from imprecise cleavage of miRNA and pre-miRNA precursors by the ribonucleases Dicer and Drosha[22]. Using this high-resolution quantitative assay, we analyzed endogenously expressed miRNAs as well as miRNAs that were overexpressed from vectors. More specifically, prior to their hybridization with specific probes, the relevant miRNA and pre-miRNA fractions were electrophoresed across different distances, which are referred to here for simplicity as short and long runs, respectively. Such an approach allowed us to separate both types of RNA molecules with 1-nt resolution.

### Applications of the Method

The protocol described here can be easily adapted for a wide range of applications. miRNAs and their precursors are not the only classes of small RNAs functioning in cells. In contrast, there are many types of short (~20 nt) and somewhat longer noncoding RNAs (ncRNA) functioning across kingdoms (e.g., endogenous siRNAs, piRNAs, snoRNAs, plant tasiRNAs, and bacterial sRNAs)[23]. They often differ in size — i.e., plant miRNAs and siRNAs are generated by different types of Dicer (the DCL1 21/22-nt and DCL2 24/26-nt size classes). Since such length diversity has functional implications, a reliable analytical method to assess RNA lengths is needed. Therefore, our high-resolution northern blot would be advantageous. The protocol could also be useful for those interested in the diverse applications of RNA interference (RNAi) and miRNA technologies; for example, at times, the molecular lengths of reagents released from vectors need to be defined precisely. In addition, it can also be used wherever the intracellular distribution and stability of various RNAi, miRNA, and antisense reagents need to be determined.

### Comparison with Other Methods

All earlier reports describing improvements in the northern blot methodology were aimed at enhancing the sensitivity of the method rather than increasing the signal resolution, which is the crux of our protocol. Moreover, the previous reports were focused on miRNAs and left many unanswered questions regarding effective pre-miRNA detection. Our protocol fills this gap and provides a detailed methodology for robust detection of both miRNAs and pre-miRNAs.

With regard to northern blot detection of miRNAs, two reports available in "*Nature Protocols*" are of particular value[9,10], as well as a new northern blot-based approach presented more recently[24]. In all these reports, the sensitivity of miRNA detection was significantly increased. In the first, probes containing locked nucleic acids (LNAs), which form stronger hybrids with miRNA targets, were used[9,25,26]. In the second, standard UV cross-linking of RNAs to nylon membranes was replaced by chemical cross-linking with EDC (1-ethyl-3-[3-dimethylaminopropyl] carbodiimide)[10,27], while the third approach harnessed the advantages of LNA, EDC, and detection using digoxigenin (DIG)-labeled probes[24]. According to the protocol described by Pall and Hamilton[10], optimal hybridization of small RNAs occurs when all nitrogen bases make hydrogen bonds with the probe. This occurs when target bases are not involved in attachment to the membrane, which takes place during UV irradiation when target bases, mostly comprised of uracil, form covalent linkages with amine groups on the membrane surface. However, the chemical method works very well for smaller molecules (<40 nt), whereas larger RNAs are fixed better with the use of UV radiation. Hence, despite being advantageous in the case of miRNAs, the method itself is less efficient for pre-miRNA detection.

### EXPERIMENTAL DESIGN

The entire northern blot procedure consists of the following steps: RNA isolation, RNA electrophoresis in a polyacrylamide (PAA) gel, transfer to a nylon membrane, immobilization on the membrane, and hybridization with a specific, labeled probe. Our objective was to achieve the best possible resolution of the miRNA and pre-miRNA fractions after their electrophoresis in PAA gels, and preserve this resolution during the subsequent steps of the northern blot protocol. The step-by-step methodology, together with useful comments regarding protocol optimization, is presented in greater detail below.

### RNA Isolation

There are many reagents available from various companies (e.g., Ambion and Qiagen), as well as protocols that enable the extraction of good-quality RNAs from various sources (i.e., plant or animal tissues). Both total RNA samples and fractions enriched in low-molecular-weight (LMW) RNAs were shown to be compatible with subsequent northern blot analyses; however, there are reports suggesting that dedicated methods should be used for the extraction of LMW and total RNAs[28,29,30]. Here we used a standard TRIzol extraction method (TRI-reagent, MRC), which is the most popular and very convenient method, for the isolation of total RNA. We show that this method is sufficient for subsequent detection of miRNAs and pre-miRNAs. Moreover, we analyzed the effects of several modifications to the TRIzol method (introduced at an RNA precipitation step) and show that the modified methods do not differ much in their performance from the original method (Fig. 1). To evaluate the outcomes of the introduced modifications, we used a mix of short, synthetic oligoribonucleotides (ORNs) (17, 19, 21, 23, and 25 nt in length) to monitor the efficiency of the RNA extractions. The short ORNs were added to samples from which total RNA was isolated at the first step of the isolation procedure, namely to TRIzol-cell mixtures. Then, RNA extraction was performed either strictly according to instructions provided by the manufacturer (Fig. 1, lane 1) or with the following modifications: elongation of the RNA precipitation step with isopropanol at -20ºC overnight (Fig. 1, lane 2) and precipitation with 96% ethanol and sodium acetate (EtOH/NaAc) (Fig. 1, lane 3). The efficacy of isolation of the short RNAs was evaluated by subsequent hybridization with a probe corresponding to the sequence of the short ORNs. The outcome confirmed that all of the RNA isolation protocols tested provided excellent results for detection of small RNAs by northern blotting. Moreover, neither the original TRIzol method nor any of its modifications led to a significant loss of ORNs. In all cases, the longest ORNs were almost completely recovered (compare

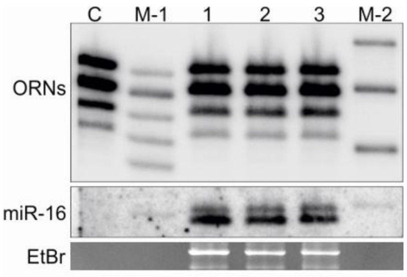

**FIGURE 1.** The impact of the RNA isolation procedure on the extraction efficiency of LMW RNAs. RNAs were isolated using either a standard TRI reagent extraction (1) or the TRI reagent method with the following modifications: prolonged precipitation with isopropanol at -20°C overnight (2) or precipitation with EtOH/NaAc (3). M denotes end-labeled size markers; 17-, 19-, 21-, 23-, 25-nt synthetic ORNs (M-1) and 20-, 25-, and 30-nt bands of LMW RNA marker from USB (M-2). Staining with ethidium bromide (EtBr) provides a loading control. Hybridization to the abundant endogenous miR-16 provides an additional control.

**Comment**: Nonlabeled, synthetic ORNs 17–25 nt in length (lane C) were run on a gel together with the samples in the quantity corresponding to synthetic ORNs extracted by the TRI method. Concentrations of the individual ORNs (17–25 nt) within their pools were equal, with the exception of the 23-nt ORNs, which were 50% more concentrated (compare with the end-labeled ORN mix of the same composition that was run as a size marker and identified as M-1). The difference in migration of ORNs between the C and M-1 lanes is caused by different end groups; not labeled oligonucleotides contain hydroxyl groups at their 5'-ends (lane C), while those labeled with [$\gamma^{32}P$] have 5'-phosphate groups (lane M-1). Such a difference in the migration rate of short RNAs (~0.5 to 1 nt) has been previously observed[22,36]. Similarly, the different migration of M-1 and M-2 markers may result from their nucleotide composition.

lanes 1, 2, and 3 with lane C in Fig. 1). However, shorter, 19-nt ORNs gave a signal that was weaker in northern blots compared to 21-nt ORNs and longer ORNs, whereas 17-nt ORNs were not detected under any of the conditions used (Fig. 1). This result may suggest that RNAs shorter than 19 nt of a similar guanine-cytosine (GC) content may escape detection by this northern blot protocol. In support of this conclusion are the results of two earlier reports that discuss the problems of probe design for the detection of closely related miRNA species[31,32].

### Electrophoretic Separation of miRNAs and pre-miRNAs

In this protocol, RNA samples are run on 12% PAA gels (19:1). Two separate electrophoreses are performed to achieve the required resolution in the miRNA fraction (~20–25 nt) and the pre-miRNA fraction (~50–70 nt). Samples are run until xylene cyanol dye (XC) migrates a distance of 10 cm, and samples for the pre-miRNA analyses are electrophoresed until XC migrates a distance of 30 cm. In each gel, the appropriate molecular weight markers are run, which are adjusted to match the lengths of the miRNA and pre-miRNA variants (Fig. 2). Precise estimation of RNA length using suitable markers is particularly important in the analysis of pre-miRNA variants. The base composition of the markers used also has to be taken into account because it may influence the rate at which markers migrate in the gel (Fig. 2, compare B and C). To evaluate the length of the pre-miRNAs precisely and rule out any possible misinterpretations, three pairs of radiolabeled ORNs with the same core sequence, but differing in length by 1 nt (75/74 nt, 64/63 nt, and 61/60 nt), were electrophoresed as described above. These ORNs were electrophoresed over three different distances to monitor the resolution of RNAs of similar lengths. Importantly, ORNs of approximately 60 nt in length separate with 1-nt resolution when the XC dye runs 20 cm, but ORNs of approximately 70 nt in length are satisfactorily separated only after the XC migrates at least 30 cm (Fig. 2C).

### Transfer of RNAs: Choice of Membrane

The RNA electrophoresis performed for either miRNAs or pre-miRNAs is followed by transfer to positively charged nylon membranes. We routinely use a semi-dry electroblotter (Sigma-Aldrich), however, a wet electrotransfer works equally well. Although voltage conditions have to be controlled carefully, the choice

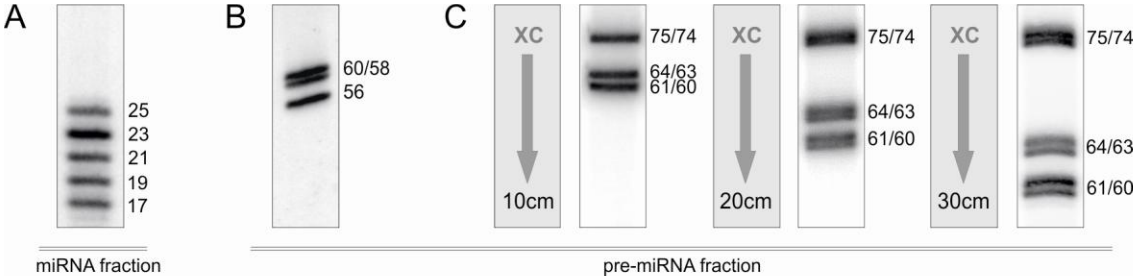

**FIGURE 2.** Electrophoretic separation of miRNAs and pre-miRNAs. (A) Resolution required for miRNA analyses. End-labeled, synthetic ORNs (17–25 nt) were run as a marker on 12% PAA until the xylene cyanol dye (XC) reached a distance of 10 cm. (B and C) The resolution required for pre-miRNA analyses. Labeled ORNs of ~60–70 nt, as indicated in the figure, were run for 10-, 20-, and 30-cm distances. Sequences of all ORNs are listed in a supplementary table (Table S1).

of membrane seems to be crucial. In accordance with previous reports[33] (http://web.wi.mit.edu/bartel/pub/protocols/miRNA_Nrthrns_Protocol.pdf), we observe differences in both signal quality and strength depending on the membrane used. We recommend using GreenScreen Plus (Perkin Elmer) or Hybond-N+ (Amersham Biosciences) membranes for the detection of miRNAs or other ~20-nt RNAs, as well as for the detection of pre-miRNAs or other RNAs of equivalent lengths. Zeta-Probe (BioRad) is also useful provided that the signals are strong enough (i.e., signals from miRNAs generated by overexpression), but it appears to be less sensitive to weaker signals typically observed from endogenous miRNAs (Fig. 3).

### Cross-Linking of Membrane RNAs

Immobilizing RNAs on membranes can be achieved by UV irradiation, baking in an oven at 80°C, or by chemical cross-linking[10]. All of these methods are efficient, but in terms of signal resolution, the chemical approach seems not to be the best choice (Fig. 4). The separation of RNAs is affected by several factors, but it is mainly influenced by the conditions of electrophoresis. A different electrophoresis buffer should be used if EDC cross-linking is to follow. The use of an EDC-compatible buffer results in compromised resolution of short RNAs. Since maximum resolution is our top priority, we recommend using UV cross-linking (120 mJ/cm$^2$), followed by baking membranes in an oven at 80°C for 30 min.

### Hybridization Conditions: Choice of Buffer

In the final step, the membranes with fixed RNAs are incubated with specific, radiolabeled DNA probes. In short, we use hybridization conditions empirically established by others[33,34,35], but here we have verified that these conditions are appropriate for detailed analyses of miRNA and pre-miRNA lengths. Of all the factors involved in determining the hybridization efficiency that we have examined, the composition of the hybridization buffer is of special importance. However, other factors, such as labeling efficiency, the kinase used for labeling, probe type, and probe purification, should also be strictly controlled. We analyzed four different hybridization buffers varying in SSC, SSPE, formamide, and SDS content (see *Reagent Setup*). We have also used a commercially available buffer (PerfectHyb, Sigma), which has been optimized to yield maximum signal. We have observed only slight differences in terms of the signal resolution and signal strength for some of the samples (e.g., miR-191), and quite substantial differences with other samples (e.g., miR-496). The latter example might be of special interest because the signal strength is different for miRNAs and pre-miRNAs depending on the buffer used (Fig. 5). Hence,

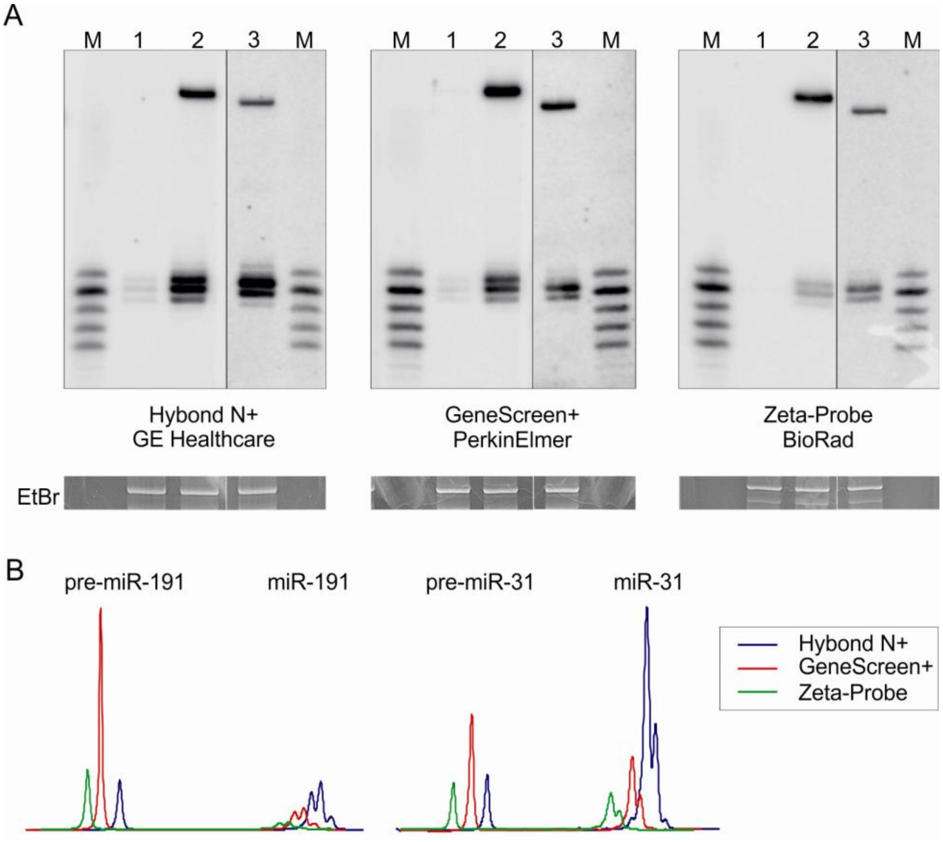

**FIGURE 3.** Transfer of RNAs: choice of membrane. (A) Hybridizations with probes complementary to miR-191 (lane 1 - endogenous expression, lane 2 - miRNA overexpressed from a vector) and miR-31 (lane 3 - overexpressed miRNA). Three different nylon membranes were used as indicated (see how one type of membrane is better for larger species, whereas the other performs better for the smaller species). M denotes a size marker; end-labeled 17-, 19-, 21-, 23-, and 25-nt synthetic ORNs. Staining with ethidium bromide (EtBr) provides a loading control. (B) Quantitative representations of the miRNA and pre-miRNA length variants are shown using peaks obtained from phosphorimaging analyses.

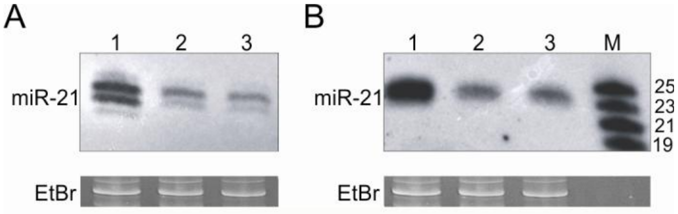

**FIGURE 4.** Cross-linking of RNAs onto membranes. Hybridization of probes to endogenous miR-21 following UV cross-linking (A) and EDC cross-linking (B). Numbers 1, 2, and 3 denote the RNAs extracted from different cell lines. M denotes a molecular size standard (19-, 21-, 23-, and 25-nt ORNs). Staining with ethidium bromide (EtBr) provides a loading control.

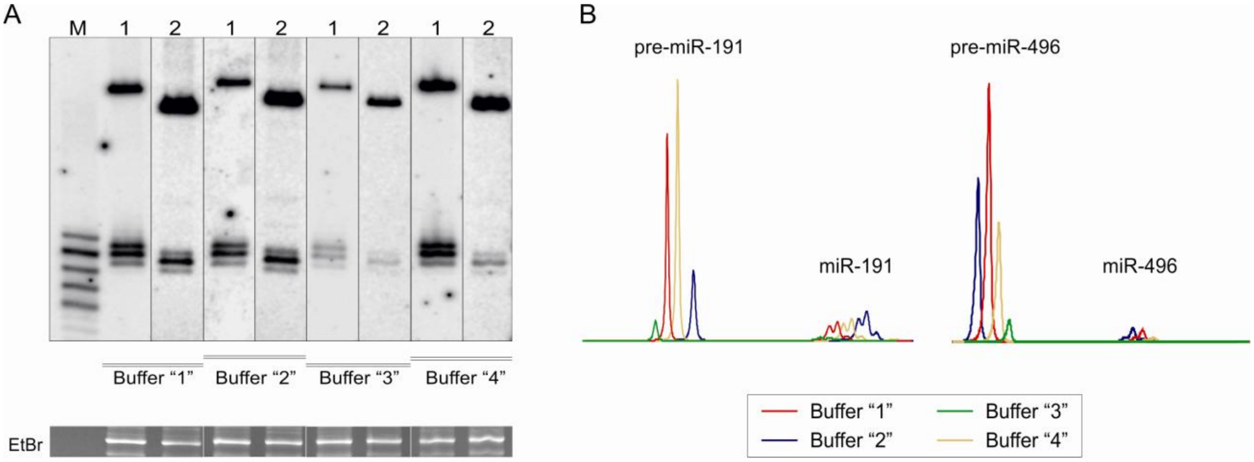

**FIGURE 5.** Hybridization conditions: choice of buffer. (A) Hybridizations with probes complementary to miR-191 (lanes 1) and miR-496 (lanes 2) in different buffer conditions as indicated in the figure (see buffer composition in *Reagent Setup*). M denotes a size marker; end-labeled 17-, 19-, 21-, 23-, and 25-nt ORNs. Staining with ethidium bromide (EtBr) provides a loading control. (B) Quantitative representations of the miRNA and pre-miRNA length variants obtained from phosphorimaging analyses.

the sequence of the probe, especially its GC content, must be taken into consideration when choosing the appropriate hybridization buffer, and both the formamide concentration and hybridization temperature should be properly adjusted. We do not recommend any specific hybridization conditions that are suitable for all purposes, but we stress the fact that these conditions may severely influence the anticipated result. Therefore, the choice of the buffer should be of prominent concern to ensure consistency of results, especially when subsequent quantification and/or comparison of northern blot signals are planned.

### MATERIALS

### 1. Reagents

- TRI Reagent (MRC, Inc., BioShop, Cat. No. TR 118) **CAUTION!** Contains toxic phenol and the irritant guanidine thiocyanate. Take appropriate safety precautions when handling. Causes burns.
- Acrylamide and bis-acrylamide (BioShop, Cat. No. ACR001.1, BIS001.250) **CAUTION!** Neurotoxic. Wear a lab coat and gloves when handling, as well as be careful not to inhale the powder.
- Urea (Chempur, Cat. No. 116615309)
- Tetramethylethylenediamine (BioShop, Cat. No. TEM001.25)
- Ammonium persulfate (Sigma, Cat. No. A3678)
- TRIS (Bioshop, Cat. No. TRS001; USB, Cat. No. 75825)
- Boric Acid (Chempur, Cat. No. 115313604)
- EDTA (ethylenediaminetetraacetic acid) (POCH, Cat. No. 71-879810112)
- SDS (20% solution) (USB, Cat. No. 75832)
- SSC (20X solution) (USB, Cat. No. 19629)
- SSPE (20X solution) (USB, Cat. No. 75890)
- Denhardt's solution (50X solution) (USB, Cat. No. 70468)
- Formamide (POCH, Cat. No. 432200116)
- Bromophenol blue (Fluka, Cat. No. 18030)

- Xylene cyanol FF (Fluka, Cat. No. 95600)
- Ethidium bromide (EtBr; Merck, Cat. No. 111608.0030) **CAUTION!** Intercalating agent, carcinogenic and mutagenic. Wear a lab coat and nitrile gloves, and take appropriate safety precautions.
- γ[$^{32}$P] ATP (Hartman Analytics, 5000 Ci/mmol, 10 mCi/ml) **CAUTION!** Appropriate safety precautions must be followed when handling radioactivity. Wear a lab coat, gloves, and eye protection.
- Nylon membrane (GeneScreen Plus Hybridization Transfer Membrane, PerkinElmer, Cat. No. NEF1017001PK; Amersham Hybond-N+, GE Healthcare, Cat. No. RPN203B; Zeta-Probe Blotting Membranes, Bio Rad, Cat. No. 162-0159)
- Whatman 3MM filter paper
- Illustra MicroSpin G-25 columns (GE Healthcare, Cat. No. 27-5325-01)
- PerfectHyb Plus hybridization buffer (Sigma, Cat. No. H 7033)
- LMW RNA Marker 10–100 nt (USB, Cat. No. 76410)

### 2. Reagent Setup

**NOTE!** Be careful while working with RNAs; ensure that all solutions and reagents are nuclease free and always wear gloves.

- PAA gel: 12% acrylamide/bis 19:1, 0.5X TBE, 7 M urea
- Electrophoresis buffer: 0.5X TBE
- Hybridization buffer “1”: PerfectHyb Plus (Sigma)
- Hybridization buffer “2”: 5X SSC, 1% SDS, 1X Denhardt’s
- Hybridization buffer “3”: 5X SSPE, 0.5% SDS, 5X Denhardt’s, 50% formamide
- Hybridization buffer “4”: 5X SSPE, 0.2% SDS, 5X Denhardt’s, 25% formamide
- Wash buffer: 2X SSC, 0.1% SDS
- Stripping buffer: 0.2X SSC, 0.1% SDS
- 2X RNA loading dye: 0.1% bromophenol blue, 0.1% xylene cyanol, 1 mM EDTA, and 95% formamide
- γ[$^{32}$P] end-labeled RNA markers; synthetic oligoribonucleotides (ORNs: 17, 19, 21, 23, 25, 56, 58, 60, 61, 63, 64, 74, 75 nt) and LMW RNA Marker 10–100 nt (USB). The sequences of all synthetic ORNs (Metabion) are provided as supplementary data (Table S1).

### 3. Equipment

- Vertical electrophoresis gel system (PROTEAN II xi Cell with gel plates 16 × 20 cm, Bio Rad) for resolution of miRNAs, and model S2 sequencing gel electrophoresis apparatus (Gibco, Life Technologies) for pre-miRNA separations
- Hybridization oven (ProBlot, Labnet)
- Cross-linker (CL-1000 Ultraviolet Crosslinker, UVP)
- Semi-dry electroblotter (Sigma-Aldrich)
- Liquid scintillation counter (Hidex)
- NanoDrop ND 1000 spectrophotometer (NanoDrop)
- Fluorescent image analyzer FLA-5100 (Fujifilm)
- Fujifilm BAS 2025 and 4043 IP cassettes
- The image analyzer software Multi Gauge v3.0 (Fujifilm)

## PROCEDURE

### Extraction of RNA (~1.5 h)

1. Isolate total RNA using TRI reagent following the manufacturer's instructions. **COMMENT:** RNAs can be stored at -20 or -80°C for shorter and longer periods, respectively, according to general RNA storage recommendations.
2. Measure RNA concentration. **NOTE!** The best concentration for northern blots is about 3 µg/µl because a higher concentration may disturb RNA separation and a lower one may necessitate loading excessive volumes of the samples.

### Gel Preparation (~1.5 h)

3. Clean glass plates and rinse them with EtOH. Prepare a gel. We recommend 12% PAA gels (19:1) of 1.5 mm in thickness. We use 1.5-mm spacers and a 10 × 1.5-mm 10-well comb (PROTEAN II gel system from BioRad). The thickness of the gel influences RNA separation and transfer, but RNA bands are sharp provided that the samples are loaded in small volumes and are loaded carefully in terms of their expelling.
4. Pour the gel and allow it to polymerize for 1 h. Assemble the electrophoresis apparatus. An aluminum plate is helpful because it ensures proper heat distribution during the electrophoresis run. **COMMENT:** Polymerized gels can be left inside the apparatus filled with 0.5X TBE buffer if they are to be used soon. Alternatively, gels can be wrapped in foil to prevent them from drying and left overnight at 4°C.

### Electrophoresis (~2–3 and ~4–5 h for miRNA and pre-miRNA runs, respectively)

5. Prerun the gel at 40 mA in 0.5X TBE running buffer for ~30 min.
6. Prepare 30 µg of total RNA from samples (or less, depending on miRNA abundance in the samples). Bring all samples to a volume equal to that of the lowest concentration of RNAs using $H_2O$. Maintain a minimal sample volume (although the wells of the PROTEAN II apparatus are very big, a sample volume up to 20 µl is optimal to ensure high resolution and sharpness of bands).
7. Mix RNAs with loading buffer (1:1). Denature RNAs prior to loading. Incubate the RNA samples for 3–5 min at 95°C and immediately chill them on ice.
8. Wash gel wells carefully before loading samples to remove any traces of urea. Wash the wells by rinsing them with electrophoresis buffer using a syringe with a thick needle.
9. To load samples, insert a pipette tip deep into the well and expel the liquid slowly. Use gel-loading tips to ensure that the samples are expelled to the very bottom of the wells. **NOTE!** Loading of RNA samples affects the sharpness of RNA bands in the gel. Compact loading of small volumes of RNAs is required to ensure the desired resolution.
10. Be sure to include at least one lane of appropriate molecular weight markers. **NOTE!** RNA and DNA markers migrate differently, and sequence composition is also important. To precisely evaluate sizes of different RNAs, use markers composed of the same core sequences that differ by one nucleotide.
11. Perform electrophoresis at 20 mA until RNA samples enter the gel and then increase the current to 30–40 mA (<600 V) in 0.5X TBE buffer until the XC dye has travelled a distance of 10 cm for miRNA gels and about 30 cm for pre-miRNA gels. Shorter runs result in insufficient separation of pre-miRNAs.

### Transfer (~1 h 15 min)

12. Prior to transfer, stain the gel with EtBr (0.5 µg/ml) to visualize rRNAs and tRNAs with UV light. Although this is not a necessary step, it helps to keep the experiment under control. Minimize exposure of RNA to UV light. Staining with EtBr does not affect northern blot quality.
13. Cut a nylon membrane (we recommend GeneScreen Plus or Hybond-N+; see the paragraph *Transfer of RNAs: Choice of Membrane* and Fig. 3) and six sheets of 3MM Whatman filter paper to the size of the gel. Presoak the membrane in 0.5X TBE. Dampen and put three filter papers on a blotter followed by the soaked membrane (anode), the gel and three wet filter papers on the top. Always wear gloves while working with the blotting membranes. Handle the membranes carefully by their edges or by using clean, blunt-ended forceps. **NOTE!** Remove air bubbles between the membrane and the gel by gently rolling a pipette several times back and forth over the surface.
14. Set the power supply of the blotter to mAmps (3 mA/cm$^2$) and limit the voltage to 20 V.
15. After 1 h, disassemble the blotting device. The gel can be stained again in EtBr to check that the transfer was efficient. All RNAs <200 nt should be fully transferred. **NOTE!** Mark the RNA side of the membrane.

### Cross-Linking (~45 min)

16. To fix the RNA by UV cross-linking, expose the side of the damp membrane with the RNA to UV light (120 mJ/cm$^2$). **NOTE!** The side of the membrane with RNA must be exposed to UV irradiation. UV cross-linking may be followed by baking membranes in an oven at 80°C for 30 min. **COMMENT:** At this stage, the membrane can be stored between two pieces of Whatman paper.

### Probe Labeling (~1 h 10 min)

17. Prepare the probe by 5'-end labeling 10–15 pmols of template DNA oligonucleotide with 50 µCi of $\gamma$-$^{32}$P with a ~1-h kinase reaction. We use OptiKinase according to the manufacturer's instructions (USB), but other kinases can be used as well (e.g., PNK). The use of OptiKinase ensures the same labeling efficiency regardless of the probe sequence. The use of a DNA probe complementary to the miRNA of interest works well in the detection of both miRNAs and pre-miRNAs. **NOTE!** Note on radioactivity ($\gamma$-$^{32}$P from Hartman Analytics incorporates well).
18. Purify the probe. We use spin columns with Sephadex G-25 (Illustra MicroSpin, GE Healthcare) since a small exclusion limit is necessary. Using such a purification method is very fast and convenient. It is done by centrifugation of the G-25 columns in a benchtop microcentrifuge according to the protocol provided by the manufacturer (GE Healthcare). Measure the probe in a scintillation counter; at least $10^6$ cpm/1 ml of the hybridization buffer is required. **NOTE!** The probe must be labeled at high specific activity ($\geq 10^8$ cpm/µg template). Too much template used for labeling will lower the specific activity of the probe.

### Hybridization (Overnight)

19. Place the membrane RNA-side-up in a hybridization bottle and prehybridize by rotating for 1 h at 37ºC in an appropriate buffer. We recommend 5X SSC buffer with 1% SDS or commercial PerfectHyb (Sigma) because they are the most effective in most cases (see Fig. 5). We use the same buffer for both prehybridization and hybridization.

20. Denature your probe by 5-min incubation at 95ºC and add to the hybridization buffer. It is not an obligatory step in the case of DNA probes, but we recommend denaturation of any kind of probes used. Hybridize overnight by rotating at 37ºC.

### Washing (~1 h 15 min)

21. Pour off the hybridization solution and wash the membrane three times with 2X SSC and 0.1% SDS for 20 min at the hybridization temperature. If necessary, continue washing at a higher temperature and/or under more stringent conditions (e.g., 1X SSC or 0.5X SSC at 42–50ºC). **CAUTION!** The hybridization solution contains radioactive probe. Discard it following appropriate safety guidelines.
22. Seal the membrane in a plastic bag or cover it with plastic wrap.

### Exposure

23. Expose the blot to a phosphor screen for at least 2 h. More often, longer exposure is necessary. We usually expose blots overnight. Autoradiography may also be performed.

### Stripping and Reprobing

24. The membranes can be reprobed several times. The successful stripping of probes is usually obtained by incubating the membrane in 0.2X SSC and 0.1% SDS at 80°C for 30 min. Always check on a phosphor screen and, if necessary, repeat the stripping or use more stringent conditions (i.e., boil the membrane in 0.1% SDS).

**TIMING:** Steps 1 and 2, 2 h; steps 3–11, 4–7 h; steps 12–16, 2 h 15 min; steps 17 and 18, 1 h 10 min; steps 19 and 20, overnight; steps 21 and 22, 1 h 15 min; step 23, 2 h to overnight.

**TROUBLESHOOTING GUIDE:** Table 1 lists some possible problems that may arise during the northern blot analysis and possible solutions.

### ANTICIPATED RESULTS

A high-resolution northern blot analysis performed according to this protocol might be especially beneficial to all applications in which the very precise separation of RNA molecules with slightly different lengths is required. The signal quality, obtained by following the procedure, is very good in terms of both the intensity and resolution. Figs. 3 and 5 demonstrate the expected results from analyses of individual miRNA length variants. Fig. 6 shows the northern blot analysis with single-nucleotide resolution of pre-miRNA length variants. The miRNA precursors generated from vector constructs were electrophoresed at the appropriate distances (see the paragraph entitled *Electrophoretic Separation of miRNAs and pre-miRNAs* and Fig. 2). Proper resolution of the pre-miRNAs enables the evaluation of length heterogeneity (Fig. 6), which results from inaccurate cleavage by Microprocessor complexes or from additional modifications.

**TABLE 1**

| Problem | Possible Reasons | Solution |
|---|---|---|
| "Smiley gel" | - Current is too high<br>- Warming of gel | Run gel at lower temperature<br>Use an aluminum plate or a suitable device to ensure proper heat distribution. |
| Insufficient resolution | - Run is too short | Small gels are enough to separate miRNAs, although the resolution is very poor and bands are not sharp. The resolution of pre-miRNAs is even more demanding and requires that XC runs for 30 cm (see Fig. 2 and step 11 of the procedure). |
| | - Volume of RNA samples is too high<br>- RNA concentration is too high<br>- Gel wells are too narrow | For optimal resolution, RNAs should be at a concentration of 3 µg/µl and the total volume should be reduced to a minimum (see *Extraction of RNA* in step 2 of procedure). |
| Weak/absent signal | - Small amount of RNA loaded<br>- No expression in samples | Load at least 30 µg RNA.<br>Hybridize to more abundant miRNAs.<br>Use an internal control (load synthetic oligos at different concentrations, starting from 0.1 pmol). |
| | - Problem with the transfer | Use a transfer control (load synthetic labeled oligos and check the intensity of their signals) . |
| | - Problem with the probe | Improve labeling efficiency; use different kinase or radioactivity from another source; increase exposure time. |
| High background, spots difficult to wash away | - High background | Try different hybridization or washing conditions (probe, stringency).<br>Use a different hybridization buffer. Commercially available PerfectHyb Plus (Sigma) yields maximum signal with minimum background in most applications. |
| | - Blots not washed enough, SDS dried onto the blots | Repeat washing, increase the washing temperature and/or stringency.<br>Wrap in a new bag, make sure the blot is wet and sealed well.<br>The use of the PerfectHyb Plus buffer (Sigma) prevents spots.<br>Pay close attention to proper purification of probes.<br>Be careful when adding your probe, do not spike the membrane with radioactivity. |
| Problem with the stripping of probes | - Membranes dried out | After cross-linking, a dry membrane can be kept at RT while membranes after hybridization should be stored at a lower temperatures to prevent undesired drying. |
| | - GeneScreen membrane binds probes strongly | Try to use different membrane. |

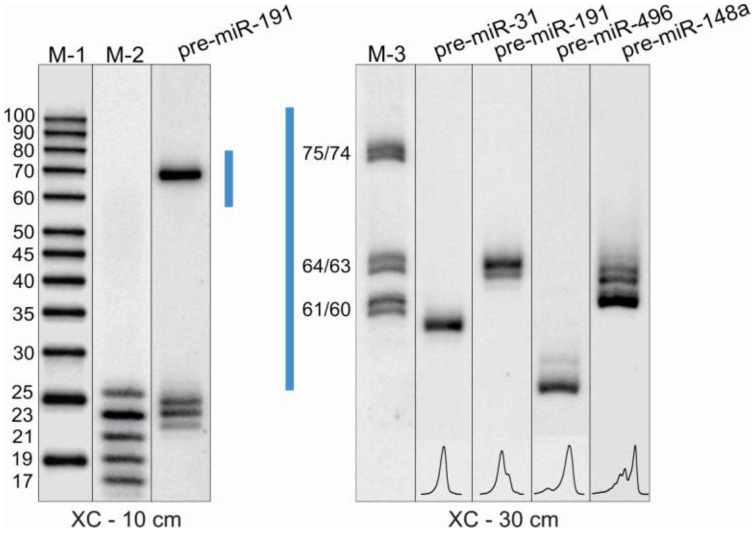

**FIGURE 6.** Anticipated resolution of pre-miRNA detection. Examples of pre-miRNAs detected with single-nucleotide resolution after short (A) and long (B) electrophoretic runs. The pre-miRNAs are indicated in the figure. M denotes the size markers; LMW RNA marker from USB (M-1), 17-, 19-, 21-, 23-, 25-nt ORNs (M-2), and 61/60-, 63/64-, 74/75-nt ORNs (M-3). The corresponding length ranges for the short and long electrophoretic runs are schematically marked as blue lines. Quantitative representations of pre-miRNA variants obtained from phosphorimaging analyses are also shown schematically.

### ACKNOWLEDGMENTS

This work was supported by the European Regional Development Fund within the Innovative Economy Programme (POIG.01.03.01-30-098/08) and by the Ministry of Science and Higher Education (N N301 523038).

### AUTHOR CONTRIBUTION

EK designed and performed experiments, implemented the protocol, and wrote the paper. JSR and KC performed experiments. WJK conceived the study, supervised the research, and contributed in the preparation of the paper.

### SUPPLEMENTARY TABLE

**TABLE S1**
**Sequences of the Synthetic ORNs used as RNA Size Markers**

| Sequence (5`→3`) | Length (nt) |
|---|---|
| GCUUAUAAAUACUGCGG | 17 |
| GCUUAUAAAUACUGCGGUC | 19 |
| GCUUAUAAAUACUGCGGUCAA | 21 |
| GCUUAUAAAUACUGCGGUCAAUU | 23 |
| GCUUAUAAAUACUGCGGUCAAUUAA | 25 |
| ACUCCAUUUGUUUUGAUGAUGGAUUCUUAUGCUCCAUCAUCGUCUCAAAUGAGUCU | 56 |
| ACCGUGGCUUUCGAUUGUUACUGUGGGAACUGGAGGUAAGAGUCUACAGCCAUGGUCG | 58 |
| ACUGUUGCUAAUAUGCAACUUUGUUGAAUAUAAAUUGGAAUUGCACUUUAGCAAUGGUGA | 60 |
| ACUGUUGCUAAUAUGCAACUCUGUUGAAUAUAAAUUGGAAUUGCACUUUAGCAAUGGUGAC | 61 |
| AGACCAUGGGUUCUCAUUGUAAUAGUGUAGAAUGUUGGUUAACUGUGGACUCCCUGGCUCUGU | 63 |
| AGACCAUGGGUUCUCAUUGUAAUAGUGUAGAAUGUUGGUUAACUGUGGACUCCCUGGCUCUGUC | 64 |
| GUUGGCUCGGGCUCCCCACUGCAGUUACCCUCCCCUCGGCGUUACUGAGCACUGGGGGCUUUCGGGCUCUGCGU | 74 |
| GUUGGCUCGGGCUCCCCACUGCAGUUACCCUCCCCUCGGCGUUACUGAGCACUGGGGGCUUUCGGGCUCUGCGUC | 75 |
